# Evaluating reliability and validity of the modified radiographic union scale for tibia (mRUST) among North American and Tanzanian surgeons

**DOI:** 10.1097/OI9.0000000000000093

**Published:** 2020-12-23

**Authors:** Alexander Coburn, David Shearer, Patrick Albright, Syed Ali, Heather J. Roberts, Billy Haonga, Edmund Eliezer, Kevin Chu, Saam Morshed

**Affiliations:** a University of California, San Francisco, San Francisco, CA, USA; b Muhimbili Orthopaedic Institute, Dar es Salaam, Tanzania.

**Keywords:** EQ-5D, mRUST, tibia

## Abstract

**Objectives:**

To determine the international reliability and validity of the modified Radiographic Union Scale for Tibial fracture (mRUST) scoring method for open tibial shaft fractures based on ratings of radiographs by separate groups of North American and Tanzanian surgeons.

**Methods:**

Seven North American and 9 Tanzanian surgeons viewed 100 pairs of AP and lateral radiographs of open tibial shaft fractures obtained in Dar Es Salaam, Tanzania. The radiographs showed 25 patients’ fractures at 4 time points postfracture after treatment with either external fixation or intramedullary nailing. Surgeons evaluated each fracture using the mRUST scoring method and indicated their confidence that the fracture was healed on a scale from 1 to 10. Reliability of mRUST was determined using inter-rater agreement among North American and Tanzanian surgeons. Validity was determined via analysis of correlation between mRUST scores and EQ-5D-3L index scores at each time point postfracture.

**Results:**

mRUST scores demonstrated strong reliability overall (ICC = 0.64) as well as within each group of North American (ICC = 0.72) and Tanzanian (ICC = 0.69) surgeons. Reliability was stronger for external fixation than for intramedullary nailing cases. mRUST scores were significantly correlated with overall healing confidence at all time points and with quality of life at 6 months and 1 year postfracture. mRUST scores also correlated significantly with patients’ quality of life scores (EQ-5D index) at 6 months and 1 year postfracture.

**Conclusion:**

North American and Tanzanian surgeons exhibited strong agreement in rating open tibial shaft fractures. Using mRUST scores is a valid means of assessing radiographic healing of tibial fractures in austere environments like Tanzania.

## 1 Introduction

Approximately half a million patients suffer from tibial fractures in the US every year.^[1]^ In low- and middle-income countries (LMIC), where rates of musculoskeletal injuries are 2 to 5 times higher than in high-income countries,^[2]^ tibial fractures likely have an even larger social impact. A crucial consideration in treatment of tibial fractures is evaluation of fracture healing. However, defining and measuring healing status remains controversial. Currently, there are no universally accepted “gold standard” measures of union or nonunion.^[3]^ This contributes to significant variability among surgeons in terms of the methods they use to determine nonunion^[4]^ and how quickly they elect to perform corrective surgery for nonhealed fractures.^[5]^ The lack of consensus about healing assessment makes it difficult for physicians to make accurate and unbiased assessments of fracture healing status.^[4]^

More recently, researchers developed the standard Radiographic Union Scale for Tibial fractures (RUST)^[3]^ and modified RUST (mRUST)^[6]^ scoring tools to assess healing status of tibial fractures using radiographic analysis. These tools use the presence of bridging callus and obliteration of fracture lines on anteroposterior (AP) and lateral radiographs in order to assign a numerical value for a healing tibial shaft fracture. In the standard RUST instrument, raters are asked to score each of 2 cortices visible on both AP and lateral radiographs of a tibial fracture. Each of the 4 (total) cortices is scored based on the following guidelines: 1 = no callus, 2 = bridging callus, 3 = remodeled. For mRUST, a fourth option (“callus present”) was added to the rating scale to differentiate between nonbridging and bridging calluses. This corresponds to a rating system of 1 = no callus, 2 = callus present, 3 = bridging callus, 4 = remodeled. Scores range from 4 to 12 (for standard RUST) and 4 to 16 (for mRUST), with lower scores indicating a less healed fracture and higher scores suggesting a more advanced stage of healing.^[6]^ The reliability (agreement in scoring among surgeons) and validity (correlation between scores and patient-relevant outcomes) of RUST and mRUST have been demonstrated in several studies.^[3,6,7]^

However, important gaps in the literature remain. First, the mRUST scoring system has never been validated internationally, as studies to date have focused on North American surgeons’ assessments of fractures. Secondly, evidence linking mRUST scores to clinically relevant patient outcomes remains limited. Finally, a minimum mRUST score threshold below which fractures can be considered “not healed” with confidence has not yet been established. This lower threshold could inform surgeons’ decisions about whether or not to perform surgery. Moreover, because clinical trials typically follow nonunions as endpoints in fracture repair studies, a nonunion threshold is also useful for research purposes.

This study addresses these gaps in the literature using data from a recently completed randomized control trial in Tanzania,^[8]^ which randomized patients with Gustilo-Anderson type I-IIIA open tibia fractures to treatment with either definitive external fixation or intramedullary nailing. The purpose of the present study was to evaluate the reliability of mRUST scoring of open tibial shaft fractures between North American and Tanzanian surgeons, and to correlate mRUST scores with both patients’ health-related quality of life and surgeons’ overall assessment of fracture healing, at 4 time points after fracture stabilization. Additionally, we sought to identify upper and lower mRUST score thresholds that correspond with healed fractures and not healed fractures, respectively.

## 2 Methods

### 2.1 Recruitment of surgeons

In this study, AP and lateral radiographs of open tibial shaft fractures were evaluated by 16 experienced orthopaedic trauma surgeons practicing at major urban medical centers in either North America (n = 7) or Tanzania (n = 9). For each pair of radiographs, surgeons determined the mRUST score and provided a rating of confidence that the fractures were healed. Surgeons were recruited by email to participate in the study, which involved completing a 20-minute online survey. Data from surgeons’ assessments of radiographs and from quality-of-life surveys were then used to assess the reliability and validity of mRUST. Informed consent was obtained from all surgeons prior to their participation in the study, and the study was approved by the IRB at UCSF. The UCSF IRB # is 14-14792, PI is Dr. Saam Morshed.

### 2.2 Selection of radiographs

Surgeons evaluated 100 pairs of AP and lateral radiographs of 25 patients with Gustilo-Anderson type I-IIIA open tibial shaft fractures who had participated in a recent randomized control trial in Dar es Salaam, Tanzania.^[8]^ This repository of images from patients with fractures was selected because of the standardization and intervals of acquisition for the parent trial, and the great variance of open [versus closed] fracture healing expected at any given time of follow-up. Patients had been treated with either external fixation (n = 14) or intramedullary nailing (n = 11). For each patient, AP and lateral radiographs and quality-of-life surveys were acquired at 4 time points postfracture (6 weeks, 3 months, 6 months, and 1 year). Radiographs were included in the study based on the availability of complete data and high-quality radiographs for each patient at all 4 time points postfracture. Seventy-three patients in the study had received both AP and lateral radiographs at each time point. A picture of each radiograph was taken and uploaded electronically for evaluation. All radiographs associated with these patients were then evaluated by a 4th-year orthopaedic surgery resident (HJR) for image quality. The quality of each image was assessed as “Good,” “Poor,” or “Obstructed,” indicating that the view of the fracture was obstructed (e.g., by an external fixator bar). Data was excluded from 48 patients whose radiographic images were assessed as “Poor” or “Obstructed” at 1 or more time points. Twenty-five patients were included who had high-quality radiographs available at all 4 time points, yielding a total of 100 pairs of AP and lateral radiographs.

### Survey design

The online survey was designed and presented using Qualtrics survey software (Qualtrics, Provo, Utah). After a live tutorial on mRUST scoring, each surgeon viewed a randomly selected subset of 25 pairs of AP and lateral radiographs, presented in random order. Each pair of images was displayed on a separate page of the survey. For each pair of radiographs, surgeons were asked to evaluate the fracture with the mRUST score and estimates of confidence that fractures were healed. For mRUST scoring, surgeons rated each cortex of the fracture as “no callus,” “callus present,” “bridging callus,” or “remodeled,” with each respective choice receiving an associated score of 1–4. Aggregation of scores for the 4 cortices yielded a total mRUST score for each fracture ranging from 4 to 16. Healing confidence was evaluated by asking surgeons to rate their confidence, on an incremental scale from 1 to 10, that the fracture was “not healed” (lower anchor) or “healed” (upper anchor).

### Quality-of-life assessment

Patients in the randomized control trial completed EQ-5D-3L quality-of-life surveys^[9–11]^ at each of the 4 time points postfracture. Patients rated 5 dimensions of their health on 3-level scales (mobility, self-care, usual activities, pain/discomfort, and anxiety/depression), as well as their overall health status using a 100-point visual analogue scale. An overall index score was then calculated for each patient and time point using the “eq5d” package in R.^[12]^ Scores were adjusted for country using the package's built-in parameters for Zimbabwe, since no parameters are currently available for Tanzania in the package.

### Statistical analysis

Inter-rater reliability was assessed using intraclass correlation coefficients (ICCs) of mRUST scores. ICCs were calculated using the “ICC” package in R,^[13]^ which estimates ICCs and confidence intervals using the variance components from a one-way ANOVA while accounting for intra-rater and intra-patient measurement groupings. Results of ICC calculations were interpreted based on the work of Landis and Koch^[14]^ and following the example of Litrenta et al.^[6]^ ICC values below 0.2 were defined as “slight agreement,” 0.21–0.40 as “fair agreement,” 0.41–0.60 as “moderate agreement,” 0.61–0.8 as “substantial agreement,” and values above 0.81 as “nearly perfect agreement.”^[14,15]^ Validity of mRUST was evaluated using linear regression models to determine the correlation between mRUST scores and patients’ quality of life (EQ-5D index) scores and surgeons’ evaluations of healing status at each time point postfracture.

## 3 Results

### 3.1 Inter-rater reliability of mRUST

Results of mRUST reliability analyses, stratified by country and treatment type, are displayed in Tables 1 and 2. ICC calculations for mRUST scoring revealed substantial agreement among all surgeons (ICC = 0.64), as well as within each subset of North American surgeons (ICC = 0.72) and Tanzanian surgeons (ICC = 0.69). Surgeons exhibited substantial agreement (ICC = 0.72) in scoring fractures treated with external fixation and moderate agreement (ICC = 0.57) in scoring fractures treated with intramedullary nailing.

**Table 1:** Inter-rater reliability of mRUST, overall and stratified by country

	All surgeons		United States		Tanzania	
			
	ICC	95% CI	ICC	95% CI	ICC	95% CI
mRUST (Total)	0.64	0.56–0.72	0.72	0.64–0.79	0.69	0.61–0.76
Anterior	0.54	0.45–0.63	0.58	0.49–0.67	0.63	0.55–0.72
Posterior	0.51	0.43–0.60	0.56	0.47–0.66	0.63	0.54–0.71
Medial	0.56	0.48–0.64	0.68	0.60–0.76	0.52	0.43–0.61
Lateral	0.53	0.45–0.62	0.66	0.57–0.74	0.66	0.57–0.74

**Table 2:** Inter-rater reliability of mRUST, stratified by procedure

	All treatment types		External fixation		IM nailing	
			
	ICC	95% CI	ICC	95% CI	ICC	95% CI
mRUST (Total)	0.64	0.56–0.72	0.72	0.62–0.82	0.57	0.46–0.69
Anterior	0.54	0.45–0.63	0.67	0.57–0.78	0.42	0.31–0.56
Posterior	0.51	0.43–0.60	0.64	0.53–0.75	0.39	0.29–0.53
Medial	0.56	0.48–0.64	0.63	0.52–0.74	0.49	0.38–0.62
Lateral	0.53	0.45–0.62	0.64	0.53–0.76	0.44	0.33–0.58

### 3.2 Validity of mRUST

Table 3 shows the results of linear regression models assessing correlations between mRUST scores of radiographs and patient life quality measures (EQ-5D index scores) at 4 time points postfracture. EQ-5D index scores were found to be significantly associated with mRUST scores at 6 months (*P* = .014, r^2^_adj_ = 0.280) and 1 year (*P* < .001, r^2^_adj_ = 0.448) postfracture. However, no significant correlations were found between mRUST scores and EQ-5D index scores at 6 weeks and 3 months postfracture.

**Table 3:** Linear regressions of EQ-5D index scores vs. mRUST scores at same time point

Time postfracture	Coefficient (B)	SE	t value	*P* value	r^2^_(adj)_
6 weeks	0.012	0.016	0.754	.46	−0.022
12 weeks	0.045	0.024	1.861	.085	0.150
26 weeks	0.021	0.007	2.761	.014	0.280
52 weeks	0.022	0.005	4.245	<.001	0.448

Correlations between mRUST scores and surgeons’ reported confidence of fracture healing are displayed in Table 4. mRUST scores were significantly associated with surgeons’ evaluations of fracture healing status at all 4 time points postfracture and within each subset of North American and Tanzanian surgeons. mRUST scores explained 89% of the overall variance in “healed” confidence ratings. Based on these regression models, the average confidence estimates of “healed” status associated with each possible mRUST score were calculated and are displayed in Table 5. mRUST scores lower than 6 were associated with <20% confidence that fractures were “healed,” and mRUST scores of 14 or higher were associated with >80% confidence that fractures were “healed.”

**Table 4:** Linear regressions of “healed” confidence vs. mRUST scores

Subgroup analysis	Coefficient (B)	SE	t value	*P* value	r^2^_(adj)_
All surgeons/times	0.770	0.031	24.76	<.001	0.891
6 weeks	0.485	0.119	4.070	<.001	0.438
12 weeks	0.893	0.187	4.782	<.001	0.610
26 weeks	0.785	0.085	9.216	<.001	0.832
52 weeks	0.788	0.051	15.416	<.001	0.918
American surgeons	0.823	0.029	28.006	<.001	0.931
Tanzanian surgeons	0.721	0.042	17.089	<.001	0.802

**Table 5:** Average “healed” confidence ratings associated with each mRUST score

mRUST score	“Healed” confidence	(95% CI)
4	7.8%	(3.7%–11.8%)
5	15.5%	(11.9%–19.1%)
6	23.2%	(20.0%–26.4%)
7	30.9%	(28.0%–33.7%)
8	38.6%	(35.9%–41.2%)
9	46.2%	(43.6%–48.9%)
10	53.9%	(51.2%–56.7%)
11	61.6%	(58.7%–64.6%)
12	69.3%	(66.1%–72.6%)
13	77.0%	(73.4%–80.7%)
14	84.7%	(80.7%–88.8%)
15	92.4%	(87.9%–97.0%)
16	100%	(95.0%–100%)

## 4 Discussion

While the mRUST scoring system has previously been validated in the North American context, this study is the first to evaluate the reliability and validity of mRUST in surgeons and patients from under-resourced countries. In the present analysis, inter-rater reliability values fell within the range of “substantial” agreement, according to the criteria outlined by Landis and Koch,^[14]^ both overall and within each subgroup of North American and Tanzanian orthopaedic trauma surgeons. Reliability was higher for fractures treated with external fixation compared to those treated with intramedullary nailing. In terms of validity, mRUST scores at later time points (6 months and 1 year) postfracture were significantly associated with patients’ self-reported general health (EQ-5D index scores). Finally, mRUST scores correlated significantly with surgeons’ estimated confidence that a fracture had healed at all 4 time points postfracture.

The reliability of mRUST has previously been validated among North American surgeons and patients.^[6,15,16]^ The present results build on this prior research. Mitchell et al^[16]^ and Litrenta et al^[6]^ identified overall ICC values of 0.71 and 0.68, respectively, for mRUST scoring of lower extremity fractures by North American trauma surgeons. By comparison, the overall ICC value of 0.64 identified here suggests that the reliability of mRUST scoring is relatively stable for surgeons operating in vastly different cultural contexts. However, inter-rater reliability was slightly higher within each subgroup of North American and Tanzanian surgeons than for the overall group of raters, which hints at possible differences across medical institutions and cultures in how surgeons applied the mRUST scoring technique for this study.

Several previous studies have also investigated the reliability of mRUST across different treatment modalities. Litrenta et al^[15]^ found greater reliability in mRUST score distributions for distal femur fractures treated with intramedullary nailing (ICC = 0.74) compared with those treated with plate fixation (ICC = 0.59). Mitchell et al^[16]^ also found that tibial fractures treated with intramedullary nailing were associated with more reliable mRUST score distributions (ICC = 0.75) than those treated with external fixation (ICC = 0.62). By comparison, the results of our study indicate that mRUST scores were more reliable for fractures treated with external fixation (ICC = 0.72) compared with intramedullary nailing (ICC = 0.57). This discrepancy in findings, while unexpected, may be due in part to differential effects of image exposure levels on the visibility of radiographs depicting fractures treated by intramedullary nailing versus external fixation.

Compared to reliability measures, validity measures of mRUST have received relatively less attention in the literature. Here, construct validity of mRUST was assessed by comparing average mRUST scores for a given patient and time point with health-related quality of life as assessed by the EQ-5D score at the same time point. The EQ-5D index score is a relevant clinical outcome measure designed to assess a patient's self-reported health and wellness at various points in the recovery process. The direct link that we discovered between a reasonably “objective” clinical tool (mRUST) and self-reported patient health at 6 months and 1 year postfracture is compelling evidence of construct validity because there is currently no gold standard method of assessing the healing status of open tibial fractures. However, we also found that mRUST scores were not significantly associated with EQ-5D index scores at earlier time points (6 weeks and 3 months). This negative finding may stem from the fact that many tibial fractures, and particularly those that are open, may take more than 3 months to show radiographic signs of healing. Meanwhile, quality-of-life measures at earlier time points may depend less on fracture stability than other factors such as wound healing or regaining range of motion.

Another important finding of this study was that mRUST scores correlated significantly with surgeons’ estimated confidence that a fracture had healed at all 4 time points postfracture. These data support the content or face validity of the instrument. Furthermore, mRUST scores explained an incrementally greater proportion of variance in “healed” confidence ratings at each successive time point postfracture, suggesting a stronger association between these 2 methods of assessment of union at later time points. The confidence intervals displayed in Table 5 provide a useful reference for surgeons to estimate how mRUST scores correspond to the likelihood that a fracture is healed or not healed. Notably, an mRUST score of 14 was associated with an average confidence of 85% that a fracture had “healed.” This is comparable to a previously-reported finding that mRUST scores of 13 or higher for distal femur fractures were rated as “healed” by >90% of North American trauma surgeons.^[6]^

This study had several important limitations. First, the radiographs available in our database were of varying quality (compared with North American standards), which could have affected the consistency of mRUST scores. We addressed this shortcoming by having an orthopaedic surgery resident (HJR) exclude poor-quality images and those in which the view of the fracture was obstructed. While this method resulted in greater consistency in image quality across the stimuli, it could have introduced some degree of selection bias into our findings. Another important limitation was that many of the surgeons who evaluated the radiographs had limited prior familiarity with the mRUST scoring method beyond the brief training that was administered at the start of each survey. This limitation could have yielded lower reliability scores than might be expected if the study were administered to surgeons with more prior training in the mRUST scoring method. Finally, our sample size was limited by the number of patients (25) who received complete radiographs at all 4 time points postfracture, due to loss to follow-up.

In conclusion, this was the first study to validate the mRUST scoring method outside of the North American context. While no “gold standard” exists for evaluating the radiographic healing status of tibial fractures, our findings suggest that mRUST is reliable in diverse clinical contexts and aligns closely with patient-relevant clinical outcomes, particularly at later stages in the healing process. This work paves the way for further research into mRUST and other tools that can improve surgeons’ assessments of fracture healing in diverse and international clinical contexts.
